# The Potential of SGLT-2 Inhibitors in the Treatment of Polycystic Ovary Syndrome: The Current Status and Future Perspectives

**DOI:** 10.3390/biomedicines11040998

**Published:** 2023-03-23

**Authors:** Dejana Rakic, Vladimir Jakovljevic, Nikola Jovic, Marija Bicanin Ilic, Aleksandra Dimitrijevic, Tatjana Vulovic, Petar Arsenijevic, Jasmina Sretenovic, Maja Nikolic, Vladimir Petrovich Fisenko, Stefani Bolevich, Gala Zarkovic, Jovana Joksimovic Jovic

**Affiliations:** 1 Department of Gynecology and Obstetrics, Faculty of Medical Sciences, University of Kragujevac, Svetozara Markovica 69, 34000 Kragujevac, Serbia; 2 Clinic for Gynecology and Obstetrics, University Clinical Centre of Kragujevac, 34000 Kragujevac, Serbia; 3 Department of Physiology, Faculty of Medical Sciences, University of Kragujevac, Svetozara Markovica 69, 34000 Kragujevac, Serbia; 4 Center of Excellence for Redox Balance Research in Cardiovascular and Metabolic Disorders, 34000 Kragujevac, Serbia; 5 Department of Human Pathology, I.M. Sechenov First Moscow State Medical University, 119435 Moscow, Russia; 6 Department of Surgery, Faculty of Medical Sciences, University of Kragujevac, Svetozara Markovica 69, 34000 Kragujevac, Serbia; 7 Clinic for Surgery, University Clinical Centre of Kragujevac, 34000 Kragujevac, Serbia; 8 Department of Pharmacology, I.M. Sechenov First Moscow State Medical University, 119435 Moscow, Russia; 9 Department of Pathophysiology, I.M. Sechenov First Moscow State Medical University, 119435 Moscow, Russia

**Keywords:** polycystic ovary syndrome, treatment, oxidative stress, hyperinsulinism, hyperandrogenism, obesity

## Abstract

Polycystic ovary syndrome (PCOS) is the most frequent endocrinopathy during women’s reproductive age. PCOS is a heterogeneous disorder featuring specific cardiometabolic properties. The association between the presence of metabolic disorders and PCOS supports the claim that the regulation of glycemic status is very important in these patients. There is a wide range of therapeutic options (including those treating diabetes mellitus type 2) with potential advantages available for the management of PCOS. Sodium–glucose cotransporter type 2 inhibitors (SGLT-2is) improve glucose metabolism, reduce fat tissue, lower blood pressure, reduce oxidative stress and inflammation, and protect the cardiovascular system. Currently, the use of SGLT-2is is not widespread in PCOS therapy, although these drugs represent a promising new therapeutic approach. Therefore, it is necessary to initiate further study in order to determine more effective therapies for PCOS and investigate the effect of SGLT-2is, both as a monotherapy and in combination with other drugs. It is necessary to understand the mechanisms underlying SGLT-2is in PCOS and their effects on long-term complications, especially since the gold standard treatment for PCOS, such as metformin and oral contraceptives, do not have long-term cardioprotective effects. The effects of SGLT-2is seem to involve cardiac protection, while diminishing endocrine and reproductive abnormalities in PCOS. In the current narrative review, we examine the most recent clinical evidence and discuss the potential applications of SGLT-2is for PCOS therapy.

## 1. Introduction

Polycystic ovary syndrome (PCOS) is the most frequent endocrinopathy experienced during women’s reproductive age and the most common cause of chronic anovulation. PCOS is not a specific endocrinological disorder with a single pathogenic mechanism, but a heterogeneous disorder whose diagnosis is established by the presence of certain criteria. Once diagnosed, the treatment approach involves eliminating all systemic factors that can falsely be presented as a PCOS and addressing its symptoms and hidden risks that are a recognized consequence of this complex syndrome. PCOS can be diagnosed in one third of women with normal ovulatory cycles. The specific definition of what PCOS represents in all its manifested and hidden forms is very important because these patients have an increased risk of developing infertility, dyslipidemia, diabetes mellitus type 2 (T2DM), obesity, hypertension, cardiovascular diseases, dysfunctional bleeding, and endometrial cancer [1].

PCOS is characterized by a constellation of interrelated reproductive abnormalities including inadequate gonadotropin secretion, hyperandrogenism, chronic anovulation, and polycystic ovarian morphology. The sources of excess levels of androgens in PCOS are the ovaries and the adrenal glands. Common comorbidities of PCOS are hyperinsulinemia and centripetal obesity. About 30–60% of PCOS patients suffer from obesity and 95% of such patients have insulin resistance. Metabolic abnormalities affiliated with this syndrome are associated with impaired glucose metabolism, diabetes mellitus, irregular adipokine activity of adipocytes, irregular production of adipose tissue, and a persistent state of low-grade inflammation [1,2].

In 1990, the National Institute of Child Health and Human Development (NICHD) set the criteria for the diagnosis of PCOS: hyperandrogenism or hyperandrogenemia, menstrual dysfunction, and exclusion of other endocrinopathies that have a similar clinical presentation. ESHRE and ASRM modified this definition in 2003 in Rotterdam, according to which the PCOS diagnosis required the presence of two of the following three entities: oligoanovulation, a clinical or biochemical sign of hyperandrogenism, and ultrasound-verified cystic formations on the ovaries, i.e., the presence of 12 follicles with a diameter of 2–9 mm and an ovarian volume greater than 10 mL^3^, simultaneously excluding other endocrinopathies that may have a similar clinical manifestation. AE PCOS stated in 2006 that the diagnosis of PCOS implies hyperandrogenism or hyperandrogenemia and ovarian dysfunction (oligoanovulation or PCOS) with the exclusion of other causes of hyperandrogenism.

Although PCOS has been known for decades, little progress has been made when it comes to the therapeutic regimen, which could lead to a complete resolving of the puzzle of this syndrome. Great efforts in the scientific community have been made to find out its precise pathophysiological mechanisms and define curative therapeutic protocols. However, until now, there has been no uniform attitude regarding etiopathogenic, diagnostic, or therapeutic issues related to this heterogeneous syndrome and all its known or lesser-known manifestations. In the current narrative review, we explored the most recent clinical evidence and discuss the potential application of sodium–glucose cotransporter type 2 inhibitors (SGLT-2is), which represents a promising new therapeutic approach for PCOS management.

## 2. Current Pathophysiological Considerations of PCOS

Despite many years of effort trying to determine the exact etiopathogenic mechanism that leads to this complex condition, the etiology of PCOS still remains unclear and the pathophysiology remains multifactorial, involving genetic, epigenetic, endocrinological, metabolic, and environmental factors.

Although the mechanisms underlying this complex disorder have not yet been fully explained, there are certain epigenetic factors [3,4], including a disorder in gonadotropin secretion [5,6], inadequate maturation of follicles [7], and insulin resistance [8], which are considered the most important factors.

When it comes to the hypothalamic–pituitary–ovarian axis, there is a defect in genetically predisposed women, resulting in an increased pulsatility of the secretion of luteinizing hormone (LH) and an increased ratio of LH to follicle-stimulating hormone (FSH) (LH/FSH). An excess of LH hyperstimulates the theca cells to produce androgens, while a low level of FSH and a high level of anti-Mullerian hormone (AMH) leads to the absence of dominant follicle selection and, furthermore, to anovulation. Increased production of androgens in theca cells promotes the secretion of insulin-like growth factor 1 (IGF1) and insulin resistance reduces the secretion of the globulin that binds sex hormones (SHBG) in the liver. It is the excess of free androgens in the circulation that leads to the manifestations of hyperandrogenism.

## 3. Current Treatment Options for PCOS

Despite the progresses that have been made in unrevealing hidden PCOS aspects, particularly cardiometabolic symptoms, the treatment remains incomplete and insuficient [9]. A complete therapeutic approach should be directed to the regulation of hyperandrogenism and menstrual cycles, the induction of ovulation, the prevention of endometrial hyperplasia, and the regulation of metabolic disorders, such as insulin resistance, dyslipidemia and the regulation of body weight if the PCOS phenotype expresses obesity. PCOS, as a cardiometabolic disorder, is related to an increased risk of developing dyslipidemia, obesity, glucose intolerance [10], T2DM [11,12,13], low-grade inflammation [14], non-alcoholic fatty liver disease (NAFLD) [15,16], and metabolic syndrome [17,18]. The phenotype of PCOS, which includes centripetal obesity, abnormalities in the distribution and function of adipocytes, hyperlipidemia, and NAFLD, interfere with the development of cardiovascular diseases and chronic diseases related to adipocyte disorders [19,20,21]. Although cardiometabolic disorders are not criteria for diagnosing PCOS, they certainly affect the deterioration of the condition and its prognosis [22].

Body weight regulation has a beneficial effect for obesity in women with PCOS. Changes in lifestyle and habits, such as the regulation of body weight, as well as the implementation of exercise and eating regimens, are the core at the beginning of any PCOS treatment. Body weight loss is important as it leads to the improvement of prediabetes, diabetes, obstructive sleep apnea, and NAFLD, prevents the onset of cardiovascular diseases, and reduces mortality. Moreover, research has shown that patients with heart failure who received dapaglifozin had a lower risk of their condition worsening or death from cardiovascular disease [23].

Traditional PCOS therapy involves regulation of the menstrual cycle, hirsutism, and ovulatory dysfunction. The first line of treatment is oral contraceptives due to their positive effect on hyperandrogenism, irregularity of the menstrual cycle, and prevention of endometrial cancer. Anovulation is treated by metformin, clomiphene citrate, letrozole, or gonadotropins. The last line of defense against infertility is in vitro fertilization.

Metformin therapy and body weight loss of at least 5% improves insulin resistance, hyperandrogenism, menstrual dysfunction, and fecundability [24]. Metformin blocks the production of ATP and increases the level of AMP, which leads to an energy deficit in those cells, and the cell adapts by phosphorylating AMP, which restores the cell’s activity, increases the sensitivity of the GLUT4 transporter, and has a favorable effect on the occurrence of ovulation. However, this has not been approved as a monotherapy for the induction of ovulation in anovulatory women with PCOS [25]. Metformin also reduces gluconeogenesis in the liver by inhibiting glycogenolysis and lipogenesis and increasing insulin sensitivity in perihepatic tissue [26]. Metformin lowers the level of free testosterone and improves lipid metabolism and endothelial function [27]. There is a dilemma as to whether metformin can be used as a therapy for non-obese infertile women with PCOS. A meta study showed that with metformin therapy, there is a higher rate of conception, but the rate of spontaneous abortions is also increased. Adding clomiphene citrate to metformin therapy increases the pregnancy rate and reduces the rate of spontaneous abortions, although there is no statistically significant difference compared to monotherapy of ovulation induction with clomiphene citrate; even letrozole as a monotherapy has shown better results in such cases [28].

The regulation of insulin resistance could be the highlight of therapies for PCOS and attempts have been made to treat this syndrome by increasing insulin sensitivity. Metformin, as the gold standard, enables the loss of body weight in obese patients undergoing monotherapy, but a dose of 1000 mg per day for 25.5 weeks is required in order to perceive its effect, while gastrointestinal complications are common [29].

Reducing the body weight of obese patients with PCOS provides numerous benefits [30]. Body mass regulation reduces the occurrence of prediabetes, diabetes, dyslipidemia, fatty degeneration of the liver, cardiovascular diseases, and obstructive sleep apnea. Formerly popular drugs that increased insulin sensitivity, do not have long-term cardioprotective mechanisms and, therefore, newer treatment methods are of greater importance [31]. The favorable effect of body weight loss has led to the conclusion that antidiabetics such as glucagon-like peptide 1-receptor agonists (GLP-1RAs) can also be used in PCOS patients. In previous years, numerous studies have shown that they studied both the efficacy and safety of the use of GLP-1RAs in patients with PCOS. There is a pilot study that showed that using long-acting GLP-1RAs in one-third of obese PCOS patients resulted in a 15% loss of body weight [32]. GLP-1RAs have been shown to be effective in their injectable form, however, this causes discomfort in patients. In addition, a greater number of patients want to use oral therapy in order to avoid pain at the site of injection and possible infection at the injection site [33]. The effect of GLP-1RAs in therapy, together with metformin, in obese PCOS women was also monitored [34]. It has been shown that the combination of GLP-1RAs and metformin improves postprandial insulin secretion compared to metformin alone. Yeribeygi et al. assume that GLP-1RAs improve insulin sensitivity by highlighting potential molecular mechanisms [35]. GLP-1RAs reduce body weight, regulate the menstrual cycle, and improve hyperandrogenism even better than metformin, but they are disadvantaged due to the need for subcutaneous administration [36]. The proven effect of GLP-1RAs and SGLT-2is on metabolic processes may suggest they are suitable for the treatment of dyslipidemia, NAFLD, obstructive sleep apnea, and PCOS [37,38].

Gliptins, also known as dipeptidyl peptidase-4 inhibitors (DPP-4i), are oral antidiabetics used as a treatment for T2DM. Their effectiveness is mainly driven by the inhibition of enzymes responsible for the degeneration of GLP-1RA. Performed studies have shown that sitagliptin, the most studied DPP-4i, improved insulin sensitivity and ovarian morphology and reduced fasting blood glucose and androgen levels [39,40]. There is evidence of the importance of targeting microRNAs (miRNAs), non-coding single stranded RNA molecules, for potential PCOS therapies. miRNAs are found in PCOS and have approximately 22 nucleotides in sequence that regulate post-transcriptional gene expression. miRNAs are related to the differentiation of adipocytes and might be potential biomarkers of obesity and its associated metabolic disorders [41].

Mio-inositol (MYO), one of the 9 forms of inositol (carbocyclic sugar present in human cells) plays an essential role in insulin signal transduction. Inositol is used as a safe dietary supplement in PCOS [42]. The intake of MYO is well-tolerated, and its recommended dose is 2–4 g/MYO per day [39]. It is beneficial for the regulation of ovulation and the response to the in vitro fertilization of infertile PCOS women because MYO improves ovulation by enhancing follicular growth and oocyte maturation and regulating hormonal disorders [39].

## 4. SGLT-2 Inhibitors: Current Knowledge on Their Therapeutic Potential in PCOS

Selective SGLT-2is are drugs that play an important role in the regulation of glucose metabolism by lowering glucose levels through an insulin-independent mechanism, reducing hyperinsulinism, and improving insulin sensitivity. They stimulate the excretion of glucose through the urine by inhibiting the reabsorption of glucose in the proximal parts of the kidney tubules (up to 50%). Glycosuria reduces the level of glycaemia by one-third of its value [43].

Many studies have shown that SGLT-2is reduce the amount of fat tissue, lower blood pressure, improve glucose metabolism, reduce oxidative stress and inflammation, and protect the cardiovascular system [44]. Loss of calories and accelerated lipolysis (from catabolism) are the mechanisms by which body mass loss occurs in SGLT-2i therapy.

With a therapeutic dose, 60–100 g of glucose is excreted through the urine, directly removing glucose from the circulatory system and lowering the glycaemia value in the blood. It has been observed that a 12-month treatment with SGLT-2is reduces levels of glycosylated hemoglobin by 0.5–0.9% (5–9 mmol/mol), decreases body weight by approximately 2 kg, and lowers both systolic and diastolic blood pressure by 2.5–5.0 mmHg and 1–2 mmHg, respectively, without accelerating the heartbeat rate and with a low risk of causing hypoglycemia [45,46]. Studies have shown that two thirds of body mass loss is fat tissue loss [47]. Except for T2DM treatment, these drugs can be safely administered in patients with heart failure and a reduced ejection fraction as well as in those with chronic renal insufficiency. Although SGLT-2is are not registered for the treatment of PCOS, clinical studies are underway that support this potential therapeutic approach. This group of antidiabetics are highly useful because of their glycemic and cardiovascular effects, which are common problems in patients with this disorder [48].

The role of SGLT-2is in the treatment of PCOS has not yet been fully studied. However, their method of action may be beneficial for several pathophysiological disorders related to PCOS, including the aforementioned insulin resistance (IR), hypertension, obesity, and dyslipidemia. The action of SGLT2i inhibitors does not depend on the insulin secretion function of beta cells or IR [39,49,50].

SGLT-2is attain further reductions in blood glucose levels by increasing insulin sensitivity, increasing muscle glucose uptake, reducing hepatic gluconeogenesis, and enhancing the release of first-phase insulin pancreatic beta cells. All described processes improve the metabolic profile of patients with T2DM, including the level of lipids and uric acid in the serum, which could also be useful for patients with PCOS. It has been shown that SGLT-2is reduce the occurrence of late complications of PCOS. The mechanism of action by which SGLT-2is act on the pathophysiological changes in PCOS could be explained by the reduction of oxidative stress mediated by sodium hydrogen exchange and the inhibition of nicotinamide dinucleotide phosphate [51]. In addition, there is evidence showing that women with PCOS have an increased risk of developing atherosclerosis of blood vessels even at an early age [52]. Within this syndrome, endothelial dysfunction often occurs, which is an indicator of the atherosclerosis development, and its early treatment can prevent the onset of cardiovascular diseases [52].

There are many metabolic disorders associated with PCOS, therefore, the regulation of glycemic status is very important. There are no studies showing the superiority of metformin in reducing body mass index (BMI) or centripetal obesity [53], while GLP-1RAs have a good metabolic effect, but must be administered subcutaneously, and DPP-4i, although administered orally, has not been shown to have a cardioprotective effect [54]. Therefore, it is very important to examine new therapeutic principles that will prove to be safe and effective in regulating the metabolic changes of PCOS.

The use of SGLT-2is is not currently widespread in PCOS therapy. The 2018 International Guideline for PCOS Therapy emphasizes the importance of body weight regulation in obese PCOS patients, and anti-obesity medications (AOM) could be an optional treatment for PCOS patients with obesity after, or in conjunction with, lifestyle changes. In 2013, canagliflozin (the first SGLT-2i) was approved by the FDA for the treatment of T2DM. Until now, these agents had only received an indication for the treatment of heart failure, T2DM, and kidney failure. It has been shown that SGLT-2is can lead to body weight loss in women who do not have T2DM [55,56]. Based on the anthropometric and metabolic effects of SGLT-2is, their applications in PCOS therapy is of great interest [3], as shown in Table 1.

## 5. Discussion

To date, there have only been a few small studies that have compared the efficacy of SGLT-2is as a monotherapy or in conjunction with metformin, as shown in Table 2. A recent small clinical study compared the use of empagliflozin (EMPA) with metformin therapy for the reduction of body mass, BMI, and fat level in patients with PCOS; however, insulin resistance, lipid profile, and hyperandrogenism was not modified [56,59]. Androgen-induced increase in intrarenal ACE expression reduces EMPA indirectly by lowering blood pressure [60]. Interestingly, these effects of EMPA occurred without modification of dihydrotestosterone in the plasma or fasting plasma levels of glucose, insulin, or cholesterol. Contrary with these results, Javed and Mudaliar claimed that SGLT-2is have been proven to lower blood pressure and the level of glycosylated hemoglobin in both humans and rodents [61].

Pruett’s study from 2021 [44] is one of the few studies that examined the effect of EMPA on an animal model of PCOS mice and showed that in hyperandrogenemic mice, EMPA lowered blood pressure and the amount of fat tissue, but did not obtain adequate results in the regulation of hormonal status. The study showed that androgens increase the renal expression of SGLT-2, SGLT-4, and GLUT2, but decrease the expression of SGLT-3. There is also increased intrarenal expression and activity of ACE caused by androgens, while EMPA lowers blood pressure and reduces the amount of fat and levels of leptin in the plasma as well as blood pressure, while failing to improve IR and albuminuria.

Tan et al. [34] showed that two weeks of therapy with licogliflozin leads to the regulation of hyperandrogenism and hyperinsulinism. Licogliflozin improves metabolic parameters and lowers the level of androstenedione and DHEAS, which are testosterone precursors. A single-blinded comparative study that lasted 24 weeks proved that treatment with 10 mg of licogliflozin per day and then 2 mg per week has the same effectiveness as 2000 mg of metformin per day in reducing body weight and waist circumference. In contrast to Javed’s study, the study by Tan et al. came to the conclusion that after 2 weeks of licogliflozin administration, the level of androstenedione and dehydroepiandrosterone sulfate (DHEAS) improved, while the regulation of androstenedione (A4) was considered the key in the regulation of ovarian hyperandrogenism by reducing the activity of 17–20 lyase.

In a study in China, 53 PCOS patients used canagliflozin and it proved to be as effective as metformin on all anthropometric, metabolic, and hormonal factors [62]. Another study showed that PCOS therapy with canagliflozin is not inferior to metformin therapy [57]. That study monitored the effect of the combination of canagliflozin and metformin on anthropometric parameters, gonadotropin secretion, the menstrual cycle, and glucose and lipid homeostasis. Usage of canagliflozin combined with metformin led to a greater lowering of free testosterone, better regulation of glucose metabolism, and greater body weight loss compared to the use of metformin as a monotherapy. Therapy with canagliflozin and metformin significantly improved menstrual bleeding, as well as the level of triglycerides and lipid status. An important difference in the comparison of canagliflozin with metformin is that canagliflozin is significantly more effective at lowering the level of uremic acid and reducing gastrointestinal problems.

Binayak Sinha et al. showed that the use of dapagliflozin for 2 weeks led to significant improvements in metabolic parameters such as a body weight loss, regulation of blood glucose levels, and improved HOMA-IR. When it comes to the hormones, it led to an improvement in DHEAS values, while there was no change in the level of testosterone or SHBG [31]. DHEAS was elevated in 40–70% of women with hyperandrogenism [63]. The regulation of DHEAS achieved by SGLT-2i therapy is very useful because hyperinsulinism induced by an increase of DHEAS can be a risk factor for the development of T2DM. Lowering the levels of DHEAS leads to body weight loss, hyperinsulinism, and lower levels of free testosterone; also, the use of glucose is favored, which could be useful for breaking the vicious cycle of hyperinsulinism and hyperandrogenism, which are the basis of the pathophysiology of PCOS [31].

**Table 2:** The effects of SGLT-2is on different parameters from preclinical and clinical studies.

Population in Study	Type of Study	Duration	Weight Loss	Drugs Given and Compared	Other Remarks	Reference
Women, aged between 18 and 45 years, BMI between 25 and 38 kg/m^2^	Meta-analysis		reduction in body weight (SMD: −0.68, 95% CI −1.16 to −0.19, <0.01),		- reduced fasting plasma glucose (FPG) (SMD: −0.59, 95% CI −0.99 to −0.19, *p* < 0.01), - reduced insulin resistance as assessed with the HOMA-IR (SMD: −0.39, 95% CI −0.76 to −0.03, *p* = 0.03 - improved DHEAS levels (SMD: −0.55, 95% CI −0.94 to −0.16, *p* < 0.01).	[32]
Women, 15 PCOS patients fulfilled the Rotterdam criteria for phenotype A or B, i.e., were overweight or obese and insulin-resistant	Randomized, double-blind, phase 2 trial.	2 weeks	- mean weight 103.9 (14.49) 106.6 (22.84) 105.2 (18.68) - BMI (kg/m^2^) mean 36·8 (4.39) - 39.5 (7.74) - 38.1 (6.27)	licogliflozin 50 mg or placebo three times a day	- reduced A4 by 19% (TR_LIK066_: TR_PCB_ [A4]: 0.81; 90% CI: 0.68–0.99; *p* = 0.089) - reduced DHEAS by 24% (TR_LIK066_: TR_PCB_ [DHEAS]: 0.76; 90% CI: 0.65–0.89; *p* = 0.008). - increased SHBG by 15% (TR_LIK066_:TR_PCB_ [SHBG]: 1·15; 90% CI: 0.97–1.36; *p* = 0.173), - Hyperinsulinemia reduced by 70% by licogliflozin (highest insulin concentration [MAXI]; TR_LIK066_:TR_PCB_ [MAXI]: 0·26; 90% CI:0.20–0.34; *p* < 0.001 and area under the curve insulin [AUCI]; TR_LIK066_:TR_PCB_ [AUCI]: 0.32; 90% CI: 0.25–0.41; *p* < 0.001).	[34]
19 women, between 18 and 45 years old, BMI ≥25 kg/m^2^, diagnosed with PCOS based on the Rotterdam criteria	Randomized open-label study	12 weeks	- weight (EMPA: −1.4 ± 3.2% vs. metformin: 1.2 ± 2.3%; *p* = 0.006) - BMI (EMPA: −1.4 ± 3.2% vs. metformin: 1.1 ± 2.2%; *p* = 0.006) - waist circumference (EMPA: −1.6 ± 2.8% vs. metformin: 0.2 ± 2.1%; *p* = 0.029) - hip circumference (EMPA: −2.0 ± 3.0% vs. metformin: 1.1 ± 1.9%; *p* = 0.001), - basal metabolic rate (EMPA: −1.8 ± 2.9% vs. metformin: 0.1 ± 1.9%, *p* = 0.024) -fat mass (EMPA: −0.7 ± 4.9% vs. metformin, 3.2 ± 5.0%; *p* = 0.023)	EMPA 25 mg (*n* = 19) or metformin 1500 mg (*n* = 20) daily	- no significant changes in hormonal or metabolic parameters - fasting glucose (mmol/L)a −0.8 ± 5.8	[62]
27 women aged 18 to 45 years with PCOS and IR	A randomized, open-label, noninferiority trial	12 weeks	- reduced body weight and total fat mass and decreased triglyceride levels	canagliflozin (100 mg OD)	- lowering of HOMA-IR (least-squares mean difference −0.81% [95% confidence interval −2.13 to 0.51 - significant advantages in reducing uric acid - reduced DHEAS - improved menstrual pattern	[57]
51 overweight or obese non-diabetic PCOS women between 18 and 40 years old	Randomized controlled trial	3 months	- BMI in the CANA/MET group (*p* < 0.0001 and *p* < 0.0001, respectively) - CANA/MET - body weight (kg) 75.40 ± 8.68 d −6.66 ± 4.24 - CANA/MET group had a mean weight loss of 5.83 kg	CANA/MET group received CANA 100 mg once daily plus MET 1000 mg twice daily, while the MET group received MET 1000 mg twice daily	- improvement in menstrual cycle irregularity was detected in CANA/MET group (80.95%, 17/21 - decrease in TT in the CANA/MET group compared to MET [CANA/MET: −2.49 ± 1.55 vs. MET: −2.20 ± 1.30; (*p* = 0.0233) - CANA/MET group, the FAI decreased (*p* = 0.0457) - CANE/MET decreased in - AUCGlu [CANA/MET: −158 ± 225.4 vs. MET: 2.63 ± 180.7; (*p* = 0.0182)] - AUCIns/AUCGlu ratio [CANA/MET: −2.86 ± 5.71 vs. MET: 0.51 ± 0.61; (*p* = 0.0164)] compared with MET	[64]
4-week-old rats	Preclinical animal study	3 weeks	- − 25.08 ± 4.17 vs. 4.82 ± 5.85%, *p* < 0.0001	EMPA (10 mg/kg/day)	- increased urinary glucose excretion - increased frequency of small adipocytes around 550 μm^2^ - increased SOD2 expression in PCOS (0.41 ± 0.17 vs. − 0.34 ± 0.12, *p* < 0.001) - increased SOD2 expression in PCOS (0.41 ± 0.17 vs. − 0.34 ± 0.12, *p* < 0.001) - increase SOD1 mRNA expression in the mWAT (0.20 ± 0.15 vs. − 0.23 ± 0.11, *p* < 0.05) - lower citrate synthase activity (32 ± 5 vs. 61 ± 8 nmol/min/mg protein, *p* < 0.05)	[65]

EMPA—empagliflozin, SOD2—superoxide dismutase2 PCOS—polycystic ovary syndrome, MWAT—mesenteric white adipose tissue, BMI—body mass index, HOMA-IR—homeostatic model assessment for insulin resistance, DHEAS—dehydroepiandrosteron sulfate, A4—androstenedione, SHBG-sex hormone-binding globulin, CANA—canagliflozin, MET-metformin, TT—total testosterone, FAI—free androgen index.

Zhang et al. were the first to perform a meta-analysis by collecting data on the efficacy and safety of SGLT-2is in the treatment of PCOS. A study that compared the use of canagliflozin with metformin alone showed no changes in the menstrual cycle, level of FSH, LH, free androgen, androgen-binding globulin, A4, or lipid status. However, there was a significant difference in the reduction of free testosterone, BMI, regulation of glycemic status, and insulin secretion [58]. Zhang also demonstrated that SGLT-2is reduce lipotoxicity and improve androgen metabolism in PCOS [58]. In addition, in obese women with PCOS, canagliflozin therapy in combination with metformin compared to metformin as a monotherapy has similar effects on the menstrual cycle, body weight, and IR, but lowers the free testosterone levels more and has a better effect on glucose metabolism [58]. Therefore, Zhang concluded, after considering the beneficial effect on metabolic parameters and the cardioprotective effect, that the application of SGLT-2is could be effective in the treatment of PCOS.

A large number of studies have shown the excellent impact of SGLT-2is on reducing the risk of cardiovascular disorders. By reducing the reabsorption of glucose and sodium and causing sodium in urine and glycosuria, these drugs lead to a loss of body mass of about 1.6–2.5 kg, affecting mainly adipocytes. SGLT-2is also have a positive impact on dyslipidemia, increasing high-density lipoprotein (HDL) and lowering low-density lipoprotein (LDL) and total cholesterol levels [64]. However, not many studies investigated the effects of SGLT-2is in the setting of PCOS-induced animal models.

Pruett and colleagues applied SGLT-2is in PCOS models and found out that there was a decrease in body mass and BMI by increasing glycosuria. EMPA reduces the amount of adipose tissue by acting only on the frequency of small adipocytes, which leads to a decrease in leptin in the plasma [44]. The mitochondrial DNA of circulating leukocytes is decreased in women with PCOS [66].

Based on the knowledge that SGLT-2is reduce oxidative stress in the heart, blood, and urine of male rodents with induced T2DM, Pruett and coworkers studied adipocyte’s vorkersound color can be removed.t.mitochondrial function in a PCOS model, as well as local oxidative stress in fat tissue [67,68,69]. They concluded that the level of oxygen radicals in oocytes in PCOS models is increased in mice, and that in subcutaneous fat tissue, both mitochondrial and cytoplasmic superoxide dismutase (SOD) are decreased with increased activity of catalase [70]. EMPA increases the expression of both cytosolic and mitochondrial SOD. They found that hyperadrenogenic female rats have mitochondrial dysfunction in white adipose tissue. SGLT-2is increase the expression of mitochondrial SOD in white adipose tissue, while in visceral adipose tissue they do not have such an effect, but instead act directly on GLUT1 and GLUT4 because it has been shown that they exist in adipocytes [71]. The affinity of SGLT-2is to GLUT1 and GLUT4 is greater than the affinity to sodium and hydrogen exchange (NHE). Mitochondrial dysfunction also occurs in endothelial cells because oxidative stress occurs when induced by increased activity of TNF alpha, which increases the cytoplasmic level of sodium and leads to the formation of reactive oxygen radicals. EMPA reduces inflammation induced by oxygen radicals and reduces NHE.

Endothelial homeostasis is a target that SLGT-2i could act on as well. Pruett’s study from 2022 [70] came to the conclusion that SGLT-2is could be used in therapy together with insulin sensitizers by stating that lowering body weight with SGLT-2i therapy fixes the frequency of small adipocytes in visceral fat tissue. Various preclinical studies showed that EMPA improved left ventricular filling pressure, causing isovolumetric relaxation, bettering vascular endothelial dysfunction and cardiac superoxide stress, and also showing a good impact on the kidneys by reducing proteinuria and the renal resistivity index, thereby reducing hemodynamic stress [65,72,73].

Certain side effects can occur during SGLT-2i administration. The use of SGLT-2is is associated with a more frequent incidence of genitourinary tract infections, acute renal failure, diabetic ketoacidosis, and bone fractures. Considering that the use of SGLT-2is increases the excretion of glucose via urine, clinical studies have shown that their use increased the rate of genitourinary infections by 3 to 5 times. Genitourinary infections are mostly of fungal origin, such as candidiasis [74,75]. Use of antifungal medications is usually sufficient in female patients to combat infection, without the need to stop SGLT-2i therapy [76]. In addition, these adverse effects have been shown in elderly diabetic patients as well, considering that diabetes represents a disease with an onset of frequent genitourinary infections. Therefore, patients must be educated on the importance of maintaining personal hygiene and consuming enough water daily in order to prevent genitourinary infections. Daily intake of water is also important to prevent dehydration and risk for falls because studies such as CANVAS accentuated the relative risk of fractures after SGLT-2i administration (hazard ratio 1.26) [77]. Moreover, there have been cases reported of Fournier’s gangrene (necrotizing fasciitis of the perineum) associated with SGLT-2i usage. On the contrary, a meta-analysis that included over 69,000 patients found no increased risk of Fournier’s gangrene, and completely excluded an increased risk for its occurrence [78]. Rarely, SGLT-2is can cause ketoacidosis by increasing glucose excretion and reducing insulin secretion, leading to hyperglucagonemia, which increases the tendency to create ketone bodies [79]. In addition, an increased synthesis of glucagon, cortisol, and epinephrine can be caused by hypovolemia due to increased diuresis using SGLT-2is, which leads to lipolysis and ketogenesis [80]. Thus, the patients who are using SGLT-2is commonly feel nausea, shortness of breath, weakness, and an urge to vomit, and even in the case of normoglycemia, urine and ketones in plasma should be tested [81]. It is worth mentioning that EMPA belongs to category C of medications, so it should be avoided during fetal renal development and in the late second and third trimester because of the possibility of hypoglycemia and its complications. There are only two case reports describing its use during pregnancy with good outcomes [82]. Therefore, the effects of the use of SGLT-2is in pregnancy is still not fully elucidated.

## 6. Conclusions and Future Perspectives

Cardiometabolic problems are as prevalent in PCOS as reproductive abnormalities. These pathophysiological events, which are frequently underdiagnosed or ignored, could have a negative impact on metabolic and cardiovascular health. It is acknowledged that the insulin pathway plays a crucial role in PCOS’s metabolic dysfunction, hyperandrogenism, and reproductive failure. Both obese and lean PCOS patients may have an aggravation of hyperandrogenemia due to hyperinsulinemia. Considering that PCOS has a detrimental effect on quality of life, it is crucial to lower cardiovascular risk factors while also addressing the reproductive and endocrine complications of PCOS. While determining the cardiovascular risk factors for PCOS patients, it is important to take into account the interactions between genetic variables, environmental influences, insulin resistance, obesity, and metabolic and reproductive dysfunctions. These characteristics may be used to identify a crucial window of time when weight increases significantly contribute to the onset of PCOS and this signals the need for preventative action against metabolic and cardiovascular diseases. In this way, different doses and treatment regimens of SGLT-2is should be carefully investigated for all PCOS phenotypes. In addition, the influence of SGLT-2is on the regulation of the menstrual cycle, ovulation induction, regulation, as well as the rate of pregnancies and the number of live births should be examined. Moreover, there is a significant lack of preclinical investigation regarding SGLT-2i effects on ovarian structure and function. This type of preclinical research will provide the mechanistic base for the already confirmed beneficial effects of SGLT-2is on the anthropometric parameters of PCOS at the very least, but likely also on the hormonal and metabolic parameters. 

Various therapeutic options with potential advantages are available for the treatment of metabolic comorbidities in PCOS. SGLT2is are promising new drugs in the treatment of PCOS, however, it is certainly necessary to initiate more studies in order to determine more effective therapies for PCOS and the effect of SGLT-2is, both as a monotherapy and in addition to other drugs. Understanding the mechanisms underlying the effects of SGLT-2is in PCOS treatment and their effects on long-term complications are important, especially since metformin and oral contraceptives do not exert a long-term cardioprotective effect, while the effects of SGLT-2is seem to exert systemic and molecular effects in cardioprotection while diminish endocrine and reproductive abnormalities in PCOS. SGLT-2is are safe hypoglycemic drugs, and the loss of body mass, prevention of late PCOS complications, and improvement of hormonal status could be the place of action of SGLT-2is in PCOS management. The heterogeneity of studies using SGLT-2is in PCOS in the way of significant diversity in the dose regimen, follow-up duration, PCOS phenotypes, and laboratory testing methodologies may all have an impact on the validity of the review’s conclusions.

## Figures and Tables

**Table 1:** The effects of different SGLT-2is on anthropometric, hormonal, and metabolic parameters.

	Given Dose	Anthropometric Parameters	Hormonal Parameters	Metabolic Parameters	Other	Reference
Tan et al.	50 mg of licogliflozin for 8 months	-	DHEAS ↓ A4 ↓ SHBG ↑ Free T = DHEA =	FG, FI ↑	-	[34]
Pruett et al.	10 mg/kg/day of EMPA to the rats for 3 weeks	BMI ↓	-	HbA1c = Leptin, Triglyceride ↓ Blood pressure ↓ IR =	Angiotensin-converting enzyme ↓	[44]
Javed et al.	25 mg of EMPA for 12 weeks, compared to 1500 mg metformin for 12 weeks	BMI ↓ FG ↓	DHT = ↔ Insulin =	BP ↓ Cholesterol =	GHB ↓	[56]
CAI et al.	100 mg of canagliflozin for 12 weeks	BMI ↓	DHEAS ↓	Triglyceride↓ Total fat mass ↓	Uric acid↓	[57]
Zhang et al.	100 mg of canagliflozin once daily plus 1000 mg metformin twice daily for 3 months	BMI =	FSH = LH = Androstenedione = SHBG =	FBG = Insulin = Triglyceride = TT ↓ LDL =	Menstrual frequency =	[58]

EMPA—empagliflozin, BMI—body mass index, FG—fasting glucose, DHT—dihydrotestosterone, BP—blood pressure, GHB—gamma-hydroxybutyrate, HBA1c—hemoglobin A1c, insulin resistance, DHEAS—dehydroepiandrosterone sulfate, A4—androstenedione, SHBG—sex hormone-binding globulin, T—testosterone, DHEA—dehydroepiandrosterone, FI—fasting insulin, FSH—follicle-stimulating hormone, LH—luteinizing hormone, FBG—fasting blood glucose, TT—total testosterone, LDL—low-density lipoprotein, ↓—decrease, ↑—increase, = no change, ↔—both directions.

## Data Availability

Not applicable.
